# Single nucleotide polymorphism rs6716901 in *SLC25A12* gene is associated with Asperger syndrome

**DOI:** 10.1186/2040-2392-5-25

**Published:** 2014-03-31

**Authors:** Jaroslava Durdiaková, Varun Warrier, Simon Baron-Cohen, Bhismadev Chakrabarti

**Affiliations:** 1 Autism Research Centre, Department of Psychiatry, University of Cambridge, 18b Trumpington Road, Cambridge CB2 8AH, UK; 2 CLASS Clinic, Cambridgeshire and Peterborough NHS Foundation Trust (CPFT), Fulbourn, Cambridge CB21 5EF, UK; 3 Centre for Integrative Neuroscience and Neurodynamics, School of Psychology and Clinical Language Sciences, University of Reading, Reading RG6 6AL, UK

**Keywords:** *SLC25A12*, Asperger syndrome, Association study, Single nucleotide polymorphisms

## Abstract

**Background:**

Autism Spectrum Conditions (ASC) are a group of developmental conditions which affect communication, social interactions and behaviour. Mitochondrial oxidative dysfunction has been suggested as a mechanism of autism based on the results of multiple genetic association and expression studies. *SLC25A12* is a gene encoding a calcium-binding carrier protein that localizes to the mitochondria and is involved in the exchange of aspartate for glutamate in the inner membrane of the mitochondria regulating the cytosolic redox state. rs2056202 SNP in this gene has previously been associated with ASC. SNPs rs6716901 and rs3765166 analysed in this study have not been previously explored in association with AS.

**Methods:**

We genotyped three SNPs (rs2056202, rs3765166, and rs6716901) in *SLC25A12* in n?=?117 individuals with Asperger syndrome (AS) and n?=?426 controls, all of Caucasian ancestry.

**Results:**

rs6716901 showed significant association with AS (*P*?=?0.008) after correcting for multiple testing. We did not replicate the previously identified association between rs2056202 and AS in our sample. Similarly, rs3765166 (*P*?=?0.11) showed no significant association with AS.

**Conclusion:**

The present study, in combination with previous studies, provides evidence for *SLC25A12* as involved in the etiology of AS. Further cellular and molecular studies are required to elucidate the role of this gene in ASC.

## Background

Autism Spectrum Conditions (ASC) are a group of neurodevelopmental conditions characterised by difficulties in social interaction and communication, alongside unusually narrow interests and repetitive, stereotyped behaviour [1]. Asperger syndrome (AS) is a subset of ASC, where there is no cognitive, developmental or language delay in childhood [2]. ASC has a concordance of 31% in dizygotic twins and 88% in monozygotic twins, suggesting a partly genetic aetiology [3]. Due to the complex and polygenic nature of the condition, the exact cause of ASC is not yet fully understood. Most candidate genes currently implicated in ASC are involved in neurodevelopmental pathways, social-emotional behaviour, or sex hormonal signalling [4].

Several genes mapped also to the region 2q24-q33 have been considered as candidate genes for autism [5-7]. The solute carrier family 25, member 12 gene (*SLC25A12*) is located at 2q24. It contains 18 exons, spread over 110 kilobases (kb) [8]. *SLC25A12* is expressed primarily as 2.9- and 3.2-kb mRNA species, predominantly in skeletal muscle, heart, and brain [6,9]. It encodes a calcium-binding carrier protein, the mitochondrial aspartate-glutamate carrier isoform 1, which localizes to the mitochondria and is involved in the exchange of the aspartate for glutamate in the inner mitochondrial membrane regulating the cytosolic redox state. It enables mitochondrial oxidation of cytosolic nicotinamide adenine dinucleotide (NADH), thought to be important in providing energy for neurons in the central nervous system (CNS) [10,11].

Several studies have identified brain metabolism abnormalities in ASC (increased cytochrome c oxidase activity, increased oxidative stress) which might be a result of mitochondrial oxidative dysfunction in neural cells [9,12,13]. *SLC25A12* may play a key role in the pathways that are altered in autism and thus can be considered a candidate gene to test in ASC.

Two SNPs in *SLC25A12* (rs2056202, rs2292813) have been associated with ASC [6] and these have been replicated in an Irish sample [14]. Family-based association analyses have provided further support that genetic variants within *SLC25A12* contribute to the aetiology of ASC in the Finnish population [15]. The study, however, did not find any association for the small AS-only family subset. rs2056202 and rs2292813 were associated with restricted repetitive behaviour traits in ASC in a small sample [16]. rs2056202 was also associated with levels of routines and rituals in autism and related conditions [17]. Nevertheless, several other studies have been unable to replicate these findings [18,19].

Thus, the role of *SLC25A12* in increasing autism risk still remains unclear. Literature provides the evidence that ASC and its subset AS share some genetic factors involved in their aetiology [20]. The aim of our study is to specifically test for association between genetic variants in *SLC25A12* and AS, to replicate previously shown results and to better understand the molecular genetics of autism. This is the first study exploring rs6716901 and rs3765166 in association with AS.

## Methods

All individuals enrolled in the current study were adults of Caucasian origin from the same geographic region (the United Kingdom). n?=?117 (43 females, 74 males) with a clinical diagnosis of AS. All cases were recruited from our online database and were diagnosed with AS by independent clinicians using either the *Diagnostic and Statistical Manual of Mental Disorders, Fourth Edition* (DSM-IV, 1994) or the *International Classification of Diseases* (ICD-10, 1994) criteria. Participants in the control group were asked to complete an online version of the Autism Spectrum Quotient (AQ) test [21], which is a measure of autistic traits. A score of 32 and above is an excellent predictor of ASC [22], and the mean AQ score in the general population is 16.4 (SD?=?6.3) [21]. Control group includes (n?=?426; 321 females, 195 males) with an AQ score below 24 to ensure a balanced representation of individuals from two ends of the autistic trait continuum. Mean AQ score within the control population was 15.2, with SD 5.2. None of the controls had a clinical diagnosis of any psychiatric conditions. This study was approved by the NHS National Research Ethics Service. Consent was obtained from all participants.

**Figure 1:**
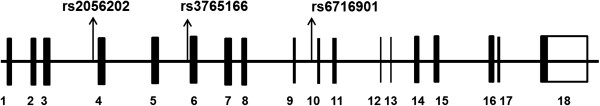
**Genomic structure of *SLC25A12 *and the location of tested SNPs.** Exons are indicated by boxes; introns are indicated by lines; SNP positions are denoted by arrows (http://www.genome.ucsc.edu).

Three SNPs (rs2056202, rs3765166, rs6716901) were selected and analysed in this study. rs2056202 has been reported to be nominally associated with ASC in the Irish and Finnish samples [14,15]. rs3765166 is a part of a common linkage disequilibrium (LD) block with rs2056202 and is a Tag SNP according to HapMap Data Release 27. Even though it belongs to the same LD block as rs2056202, genotyping a Tag SNP would be more informative, as it is in high LD with a greater number of SNPs including rs2056202. rs6716901 is not in LD with either of the two. The SNPs selected were limited by those available in the ABI TaqMan assay that was used for genotyping. All three SNPs are intronic SNPs (see Figure 1).

LD values between SNPs of interest in the HapMap CEPH European samples of the Utah Residents with Northern and Western European Ancestry (CEU) population data were calculated using SNAP (http://www.broadinstitute.org/mpg/snap/). Minor allele frequency (MAF) for the tested SNPs was above 0.05 in the CEPH CEU population as calculated from the dbSNP database (http://www.ncbi.nlm.nih.gov/projects/SNP/). DNA was extracted from buccal swabs and anonymised. SNP genotyping was performed using TaqMan SNP Genotyping Assays (Applied Biosystems Inc., Foster City, CA, USA) using a previously described protocol [4]. Allelic association testing was performed using Plink v1.07 (http://pngu.mgh.harvard.edu/~purcell/plink/) [23]. Bonferroni correction was performed to correct for multiple SNPs. Functional annotation was performed using HaploReg (http://www.broadinstitute.org/mammals/haploreg/haploreg.php) [24], SNPnexus (http://snp-nexus.org/) [25] and F-SNP (http://compbio.cs.queensu.ca/F-SNP/) [26].

## Results

In the association analyses, rs2056202 (*P*?=?0.26) and rs3765166 (*P*?=?0.11) showed no significant association with AS. rs6716901 showed significant association with AS (*P*?=?0.008). Results remained significant after correcting for multiple testing. None of the SNPs deviated from the Hardy-Weinberg equilibrium. Genotyping rate was above 98%. Data are summarized in Table 1.

**Table 1:** Single SNP association analyses

**dbSNp ID**	**Alleles**^**a**^	**MAF**^**b**^	**Odds ratio**	**Confidence interval**	**F_Ac**^**c**^	**F_U**^**d**^	**Chi-sq**^**e**^	***P*-value**^**f**^	**Alpha**^**g**^
rs6716901	G/A	0.13	1.70	0.98 to 3.02	0.18	0.11	6.87	*0.008*	0.016
rs2056202	C/T	0.13	0.76	0.39 to 1.46	0.10	0.13	1.29	0.26	0.016
rs3765166	G/A	0.23	1.31	0.81 to 2.07	0.27	0.22	2.51	0.11	0.016

Significant *P*-values are written in italics. MAF, minor allele frequency.

^a^common allele is listed first.

^b^calculated by Plink v1.07 in analysed sample.

^c^the frequency of the minor allele in cases.

^d^the frequency of the minor allele in controls.

^e^the chi-squared statistic for this test (1 df).

^f^computed on the basis of likelihood ratio test.

^g^determined after evaluating the number of completely independent SNPs using SNPSpD.

## Discussion

In this study, three SNPs (rs2056202, rs3765166, rs6716901) in *SLC25A12* were tested for association with AS. We identified an association between rs6716901 and AS in our sample. rs2056202 has been reported to be nominally associated with ASC in Irish and Finnish samples [14,15]. This association was not replicated in our sample. Some other studies reported discrepant results and found no significant association between *SLC25A12* and autism [18,19].

Inconsistency between studies can be explained by several factors. An ASC is a complex condition with considerable variation in the cognitive and behavioural phenotypes. Hence, probands recruited for genetic studies are very heterogeneous. It is likely that different research groups include cases with different clinical symptoms. To the best of our knowledge, this is the first non-family-based association study between *SLC25A12* and AS specifically. While this reduces heterogeneity, the dimensional nature of the conditions remains an issue. We demonstrated the association between rs6716901 of *SLC25A12* with AS, supporting the role of this gene in the aetiology of ASC, but *SLC25A12* might be associated with certain clinical symptoms or behavioural features of autism, rather than the diagnosis of autism itself. In other words, it may be a modifier gene, rather than a causative gene. For example, the A allele of rs2056206 of *SLC25A12* was significantly associated with lower levels of routine and ritual behaviour in autism [17].

The functional role of rs6716901 is unclear. It is not in high LD with other common variants that alter transcription or chromatin states when queried on HaploReg. There are no copy number polymorphisms or miRNA binding sites associated with the SNP when queried on SNPnexus. Analyses at a cellular level would need to be carried out to understand how this SNP can contributes to the AS phenotype. Increased activity of the mitochondrial aspartate-glutamate carrier proteins and elevated levels of *SLC25A12* have been detected in the superior temporal region of post-mortem brains of people with ASC [27]. Increased expression of *SLC25A12* transcript has also been found in the prefrontal cortex of people with ASC [28]. During foetal development, *SLC25A12* molecular gradients have been identified in the lateral prefrontal and ventral temporal cortex. These foetal structures show abnormalities in autism [28]. *SLC25A12* is also required for the synthesis of myelin lipids in brain neurons [29]. A missense mutation in *SLC25A12* leads to changed protein activity, and global hypomyelination in the cerebral hemispheres, suggesting that impaired efflux of aspartate from neuronal mitochondria prevents normal myelin formation [30]. Alternation of *SLC25A12* expression in mouse embryonic cortical neurons affects dendrite length and the mobility of dendritic mitochondria [28]. Taken together, variation in *SLC25A12* expression may be involved in the pathophysiology of autism, modifying both neuronal structures and metabolism in the CNS.

A limitation of our study is sample size. It has only limited power to reliably detect the role of certain variants in the genetics of the condition. It is worth mentioning that females are over-represented in the control sample compared to the AS group. In the sex-stratified analyses, none of the SNPs are significant after Bonferroni correction, indicating that the over-representation of females in controls is not driving the association (data not shown). Another limitation is the lack of a replication sample. According to our findings, *SLC25A12* may contribute to genetic susceptibility of autism in some populations, but further studies with larger sample size are needed to address and clarify the role of this gene in autism. Moreover, a single gene is unlikely to have a major effect in complex conditions like autism, and many other genes are likely to contribute to the phenotype.

## Conclusions

Three SNPs (rs2056202, rs3765166, and rs6716901) in *SLC25A12* were genotyped in n?=?117 individuals with AS and n?=?426 controls, all of Caucasian ancestry. rs6716901 showed significant association with AS (*P*?=?0.008) after correcting for multiple testing. The present study, in combination with previous studies, provides evidence for *SLC25A12* being involved in the etiology of ASC. Further cellular and molecular studies are required to elucidate the role of this gene in ASC.

## Abbreviations

AQ: Autism Spectrum Quotient; AS: Asperger syndrome; ASC: Autism Spectrum Conditions; CEU: European samples of Utah Residents with Northern and Western European Ancestry; CNS: central nervous system; DSM-IV: *Diagnostic and statistical manual of mental disorders fourth edition*; ICD-10: *International classification of diseases*; LD: linkage disequilibrium; MAF: minor allele frequency; NADH: nicotinamide adenine dinucleotide; SLC25A12: solute carrier family 25 (aspartate/glutamate carrier); SNP: single nucleotide polymorphism.

## Competing interests

The authors declare they have no competing interests.

## Authors’ contributions

BC and SBC co-designed the study. SBC obtained funding for the study. VW and JD conducted the analysis. JD wrote the first draft of the paper revised by all authors. All authors read and approved the final manuscript.
